# Triptolide Attenuates Transplant Vasculopathy Through Multiple Pathways

**DOI:** 10.3389/fimmu.2020.00612

**Published:** 2020-04-21

**Authors:** Zihuan Luo, Tao Liao, Yannan Zhang, Haofeng Zheng, Qipeng Sun, Fei Han, Zhe Yang, Qiquan Sun

**Affiliations:** Organ Transplantation Research Institute, The Third Affiliated Hospital of Sun Yat-sen University, Guangzhou, China

**Keywords:** transplant vasculopathy, triptolide, IFN-γ, donor-specific antibodies, vascular smooth muscle cells

## Abstract

Transplant vasculopathy (TV), a hallmark of chronic allograft rejection, is the primary cause of allograft loss after organ transplantation. Because multiple mechanisms are involved in TV pathogenesis, effective therapy for it remains elusive. Here, we identify the role of triptolide, which has a wide spectrum of immuno-suppressive activities, in inhibiting TV development. Murine aortic transplants models were constructed and divided into triptolide-treated and untreated groups. We found that triptolide significantly alleviated intima thickening of allografts by inhibiting multiple pathways. Triptolide significantly reduced infiltration of T lymphocytes and macrophages and inhibited the levels of pro-inflammatory (TNF-α, IL-2, and IL-6) and pro-fibrotic factors (TGF-β, α-SMA, and MMP-9) in the graft. Additionally, triptolide significantly decreased the numbers of IFN-γ-producing T lymphocytes, as well as the expression of IFN-γ and IFN-γ-inducing factor (*CXCL9* and *CXCL10*) in recipient. Moreover, triptolide decreased the numbers of B lymphocytes and plasma cells, as well as the levels of donor specific antibodies (DSAs) in recipient. Furthermore, triptolide not only inhibited vascular smooth muscle cell (VSMC) viability and promoted VSMC apoptosis but also significantly inhibited VSMC migration *in vitro*. These results emphasize the efficacy of triptolide in inhibiting TV development and provide a basis for developing new treatments to prevent TV-related complications and improve the long-term survival of transplant recipients.

## Introduction

Organ transplantation is an ideal and final solution for patients suffering end-stage organ diseases (1). However, approximately 90% of allografts are likely to develop transplant vasculopathy (TV) during long-term follow-up (2). TV, featured by arterial intimal hyperplasia and inflammation, is a key component of chronic allograft rejection, and the lethal factor of late allograft failure (3, 4). So far, there is no effective therapy for TV, the only definitive treatment currently available for TV is re-transplantation (5). Therefore, novel therapeutic agents based on an improved understanding of the risk factors that contribute to TV might overcome this dilemma.

It is well known that multifactorial events participate in the development of TV. Previous studies have reported inflammation as a primary mechanism of TV development (6), moreover, the formation of TV needs the involvement of the interferon (IFN)-γ axis, as TV do not occur in settings of congenital absence or neutralizing antibody blockade of IFN-γ (7, 8). Excessive activation of inflammation further leads to the development of vascular fibrosis, which promotes TV formation (9). Some reports demonstrate that antibodies are independent risk factors for long-term survival of recipients, and can cause or contribute to TV (10, 11). Additionally, vascular smooth muscle cell (VSMC) migration and proliferation are key events involved in TV development (12, 13). Given the numerous pathogenic factors associated with TV, there is no effective therapeutic strategy capable of simultaneously regulating its multiple pathogenic pathways.

Triptolide is a structurally unique diterpene triepoxide and a principal bioactive component of the Chinese traditional medicine *Tripterygium wilfordii* Hook F, which is broadly used in clinic due to its strong immunosuppressive and anti-proliferative properties (14, 15). Triptolide has been proved to suppress the proliferation and activity of T lymphocytes and macrophages (16, 17), and is a strong inhibitor of IFN-γ signaling pathway in tumors and inflammation-related diseases (18, 19). However, there are few studies exploring its effects on antibodies. Our preliminary study found that triptolide inhibited the production of antibodies in acute rejection model (20). However, whether triptolide can play the similar roles in the chronic rejection model remains to be further studied. As far as we know, triptolide has been shown to inhibit the proliferation of VSMC (21), but there is no direct evidence that triptolide inhibits migration of VSMC, especially during the formation of TV.

Given the extensive immunosuppressive and anti-proliferative properties of triptolide, we hypothesized that it might be an ideal inhibitor of TV. Therefore, we investigated the efficacy and mechanisms of triptolide in attenuating TV using a murine aortic transplant model.

## Materials and Methods

### Animals and Abdominal Aortic Transplantation Procedures

Male adult C57BL/6 and BALB/C mice (Beijing Vital River Laboratory Animal Technology Co., Ltd., Beijing, China) weighing between 20 and 25 g, were used as donors or recipients, respectively. Animals were housed in a specific pathogen-free facility at Sun Yat-sen University (Guangdong, China), and all animal experiments were performed in accordance with the Guide for the Care and Use of Laboratory Animals (National Institutes of Health publication No. 80-23, revised 1996) and according to the Sun Yat-sen University Institutional Ethical Guidelines for animal experiments. Abdominal aortic transplantation was performed using a previously described technique with modifications (22). Briefly, a 10–15 mm segment of C57BL/6 donor infrarenal abdominal aorta was isolated, resected, and replaced with the segment of BALB/C recipient infrarenal aorta with end-to-end anastomoses using 12-0 monofilament nylon sutures (Ethicon, Somerville, NJ, United States) under an operative microscope. The complete grafting procedure required 45 min to 60 min, and all surgeries were performed under inhalation anesthesia with methoxyflurane (Metofane; Pitman-Moore, Mundelein, IL, United States). Technical success was defined as grafts not becoming occluded during the first 10 days after transplantation. The graft success rate was >90%.

### Treatment Protocol

All mice were weighed before and during treatment. Recipients were randomly assigned to two groups (*n* = 5/group): the triptolide group, which was subcutaneously administered triptolide (0.5 mg/kg; Chinese National Institute for Control of Pharmaceutical and Biological Products, Beijing, China) every other day, initiating at day 0 after aortic transplant and continuing through the end of the experiment (day 28 after transplantation); the untreated group, which was subcutaneously administered an equal volume of normal saline. No other immunosuppressive medication was used.

### Graft Harvesting and Morphometric Analysis

Grafts were harvested at day 28 under anesthesia. For histomorphometry analysis, tissue cross-sections (4-μm thick) were cut, deparaffinized, and rehydrated, followed by staining with hematoxylin and eosin. The sections were examined for severity of luminal stenosis using a DMR Leica microscope (Leica, Bannockburn, IL, United States) and Image-Pro Plus (IPP) 6.0 imaging software (Media Cybernetics, Silver Spring, MD, United States) by an experienced pathologist who was blinded to the groups. The cross-sectional area of luminal stenosis was calculated using the following formula: luminal occlusion (%) = (internal elastic lamina area - luminal area)/(internal elastic lamina area) × 100. Thickness of intimal and intimal medial layers were measured from 10 sites per graft section and intima/intima + media ratios were calculated as described (23). Furthermore, luminal stenosis of the arterial graft was also determined using a previously described scoring system (24).

### Immunohistochemistry (IHC)

For IHC analysis, the cross-sections (4-μm thick) were deparaffinized and rehydrated, followed by incubation with antibodies against CD3 (Abcam, ab135372, 1:800), CD4 (Abcam, ab183685, 1:800), CD8 (Abcam, ab217344, 1:1000), and CD68 (Abcam, ab125212, 1:1000) at 4°C overnight. The samples were then stained with Goat Anti-rabbit IgG/HRP (Bioss, bs-0295G-HRP, 1:100) for 1 h at 37°C. Adventitial CD3+, CD4+, CD8+, and CD68+ cells were scored by cell counting using IPP 6.0 imaging software (Media Cybernetics) and expressed as cell number per vessel section (400× magnification).

### Real-Time Quantitative Reverse Transcription Polymerase Chain Reaction (qRT-PCR)

Levels of proinflammatory cytokine mRNA in allografts were determined by qRT-PCR. Total RNA was extracted from frozen graft tissue and mononuclear cells from recipient spleens using a homogenizer and TRIzol reagent (Invitrogen, Carlsbad, CA, United States), and cDNA was reverse transcribed using PrimeScript RT master mix (Perfect Real Time; TAKARA, Shiga, Japan). qRT-PCR was performed in triplicate using SYBR Green I master mix (Roche, Basel, Switzerland) in a LightCycler480 system (Roche), with *glyceraldehyde 3-phosphate dehydrogenase* (*GAPDH*) used as an internal control. Primers used for qRT-PCR are provided in Table 1.

**TABLE 1:** Primers for qRT-PCR.

Gene	Forward primer	Reverse primer
IFN-γ	GGAACTGGCAAAAGGATGGTGAC	GCTGGACCTGTGGGTTGTTGAC
CXCL9	ATCTCCGTTCTTCAGTGTAGCAATG	ACAAATCCCTCAAAGACCTCAAACAG
CXCL10	AGGGGAGTGATGGAGAGAGG	TGAAAGCGTTTAGCCAAAAAAGG
TNF-α	CAGGCGGTGCCTATGTCTC	CGATCACCCCGAAGTTCAGTAG
IL-2	ATGAACTTGGACCTCTGCGG	ATGTGTTGTCAGAGCCCTTT
IL-6	GATGAAGGGCTGCTTCCAAC	GCTTCTCCACAGCCACAATG
TGF-β	CTTCAGCTCCACAGAGAAGAACTGC	CACGATCATGTTGGACAACTGCTCC
α-SMA	CTGGAGAAGAGCTACGAACTGC	CTGATCCACATCTGCTGGAAGG
MMP-9	CGTCGTGATCCCCACTTACT	AGAGTACTGCTTGCCCAGGA
GAPDH	TGACCTCAACTACATGGTCTACA	CTTCCCATTCTCGGCCTTG

### Determination of Circulating Donor-Specific Antibodies (DSA)

Levels of circulating donor-specific antibodies (DSA; IgG and IgM) in recipient sera at the indicated time points were assessed by flow cytometry, as previously described (20). Briefly, recipient sera were incubated with C57BL/6 donor splenocytes at 37°C for 30 min, after which washed cells were incubated with fluorescein isothiocyanate (FITC)-labeled anti-mouse IgG (Abcam, ab6724, 1:100) and rhodamine red-conjugated anti-mouse IgM (Jackson ImmunoResearch Laboratories, 115-297-020, 1:100) at 4°C for 1 h. Cells were analyzed by FACScan (Becton–Dickinson, Lincoln Park, NJ, United States) flow cytometry with results expressed as mean fluorescence intensity to reflect individual serum DSA levels.

### Flow Cytometry

Fresh recipient spleens were milled gently in phosphate-buffered saline (PBS) supplemented with 1% heat-inactivated fetal bovine serum using a needle on a 5-mL syringe, followed by pressing through a 200-um mesh nylon screen. Mononuclear cells from whole blood or spleen of recipient were collected by Ficoll density gradient centrifugation (Solarbio, P8860). Mononuclear cells were then stained with fluorochrome-conjugated antibodies against CD3, CD4, CD8a, IFN-γ, CD45, CD19, and CD38. For intracellular IFN-γ staining, cells were first stimulated at 37°C for 5 h with a leukocyte-activation cocktail (BD, 550583), followed by staining with a surface marker, and further fixed and permeabilized using Cytofix/Cytoperm^TM^ (BD, 554714) according to the manufacturer’s instructions for intracellular staining.

To evaluate the effect of triptolide on IFN-γ-producing T cells *in vitro*, mononuclear cells from recipient spleens were seeded at a density of 1 × 10^6^ cells/well and plated into a 24-well plate (Corning Inc., Corning, NY, United States). The cells were then stimulated with anti-CD3 (Clone: UCHIT, BD Pharmingen, San Jose, CA, United States; 1 μg/ml) and anti-CD28 (clone: CD28.6 eBiosciences, San Diego, CA, United States, 1 μg/ml) with or without tiptolide (4 ng/mL) at 37°C for 24 h. Then, cells were cultured in the presence of 1 μg/mL brefeldin A (BFA, Sigma, B7651) for 12 h, harvested, followed by staining with a surface marker, and further fixed and permeabilized for intracellular staining. Data were analyzed using the FlowJo software (Tree Star, Ashland, OR, United States). Antibodies used for flow cytometric analysis were purchased from BioLegend (San Diego, CA, United States), including: FITC-CD4 (#130308,1:100), PerCP/Cy5.5-IFN-γ (#505822,1:100), BV570-CD45 (#103136, 1:100), PerCP/Cy5.5-CD19 (#115532,1:100), and FITC-CD38 (#102705, 1:100). Other antibodies were obtained from eBioscience (San Diego, CA, United States), including: eFluor 450-CD8a (#48-008, 1:100) and Alexa Fluor 700-CD3 (#56-0032, 1:100).

### Cell Culture

Aortic VSMCs (MOVAS-1) was a gift from Southern Medical University (Guangdong, China). The cells were maintained in Dulbecco’s modified Eagle’s medium (DMEM) containing 10% FBS, 100 U/mL penicillin, and 100 μg/mL streptomycin (both from Wuhan Procell Lite Science & Technology Co., Ltd., Wuhan, China) at 37°C under 5% CO_2_.

Mononuclear cells from recipient spleens were maintained in RPMI-1640 supplemented with 10% FBS, 100 U/mL penicillin, and 100 μg/mL streptomycin at 37°C with 5% CO_2_.

### Cell Viability Assay

Cell counting kit-8 (CCK-8; Beyotime Biotechnology, Shanghai, China) assays were performed to evaluate the viability of VSMC following treatment with different concentrations of triptolide. Briefly, VSMCs were seeded at a density of 1 × 10^4^ cells/well in a 96-well plate (Corning Inc.), followed by exposure to PBS or various concentrations of triptolide (0, 5, 10, 20, 40, and 80 ng/mL) for 24 h and 48 h, and the subsequent addition of CCK-8 solution (10 μl). Cells were then incubated for 1 h at 37°C and 5% CO_2_, and the absorbance of each well was recorded at 450 nm using a microplate reader (Bio-Tek, Winooski, VT, United States). Cell viability (%) was calculated as follows: (average absorbance of triptolide-treated cells/average absorbance of PBS-treated cells) × 100.

### Apoptosis Assay

The *in vitro* effects of triptolide on VSMC apoptosis were determined using the Annexin V-FITC/propidium iodide assay. VSMCs were seeded at a density of 1 × 10^6^ cells/well and plated onto a 6-well plate (Corning Inc.), followed by exposure to various concentrations of triptolide (0, 5, 10, 20, 40, and 80 ng/mL) for 24 h and 48 h, after which Annexin V-FITC/PI (Thermo Fishier Scientific, #V13242) was added to the cells and incubated for 10 min at 25°C in the dark according to manufacturer instructions. Cell apoptosis was analyzed by flow cytometry using a FACScan system (Becton–Dickinson, Lincoln Park, NJ, United States).

Mononuclear cells from recipient spleens were seeded at a density of 1 × 10^6^ cells/well and plated in a 24-well plate (Corning Inc). Following exposure to various concentrations of triptolide (0.04, 0.4, 4, 40, or 400 ng/mL) for 72 h, apoptosis assays were performed as described (20).

### Transwell Migration Assay

Vascular smooth muscle cell migration was determined using Transwell chambers, with a Transwell membrane containing 8-μm pores (Costar; Corning) and coated with 10 μg/mL fibronectin, inserted into a 24-well plate. The lower chamber was filled with 600 μL of DMEM with 10% FBS, and VSMCs (1 × 10^5^ cells/well) in serum-free DMEM were placed in the upper chamber, followed by incubation with PBS or various concentrations of triptolide (0, 5, and 10 ng/mL) for 24 h. Infiltrated cells were fixed in 4% paraformaldehyde (Bioss, C01-06002) and stained with 0.1% crystal violet (Bioss, D10162). Migrated cells were photographed under an inverted microscope (LEICA DMI 4000B, Germany), and five random high-power fields (200× magnification) were selected for quantification of cell number using the IPP 6.0 imaging software (Media Cybernetics). VSMC-migration ability was expressed as the ratio of the number of migrated cells to that of control cells.

### Statistical Analysis

Data are expressed as mean ± standard deviation (SD). Normal distribution was first used to test the distribution of data using KS normality test. All data was normal distribution. Comparisons between two groups were performed using Student’s *T*-test. Statistical analysis was performed using Prism (GraphPad). Probability values of *P* < 0.05 were considered significant.

## Results

### Triptolide Inhibits Intimal Hyperplasia in Murine Aortic Allografts

**FIGURE 1:**
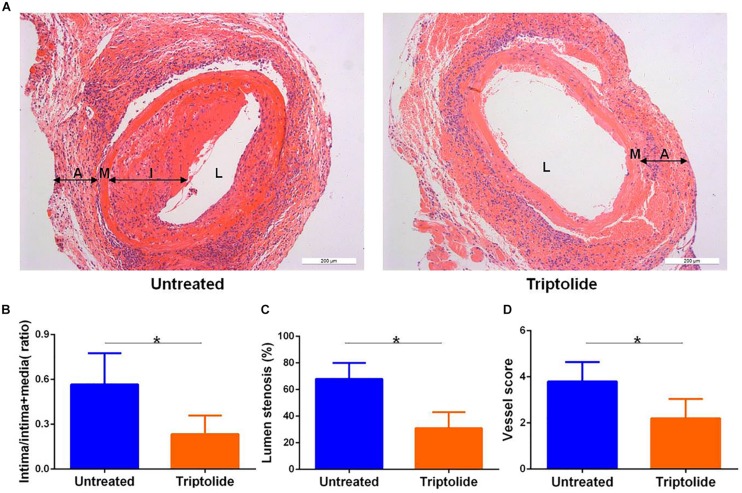
Triptolide inhibits intimal hyperplasia in murine aortic allografts. **(A)** Representative photomicrographs of hematoxylin and eosin stained whole (100×) vascular allografts 4 weeks after transplantation showing TV lesions. **(B)** Intima/intima + media ratio **(C)** lumen stenosis, and **(D)** vessel score. Data were represented as mean ± SD (*n* = 5). *p < 0.05 vs. untreated Student’s *t*-test. A indicates adventitia; M, media; I, intima; and L, lumen.

We established an allogeneic aortic graft model, and the recipients were treated with or without triptolide for 4 weeks; subsequently, allografts were harvested and tissue sections were stained with hematoxylin and eosin to visualize TV lesions. As shown in the representative photomicrographs, the arterial intima of the triptolide-treated group was observably thinner than those of untreated group (*p* < 0.05, Figure 1A). Additionally, we measured the intima/intima + media ratio (0.57 ± 0.14 vs. 0.23 ± 0.09; Figure 1B) lumen stenosis (68% ± 12% vs. 31% ± 11%; Figure 1C), and vessel score (3.80 ± 0.84 vs. 2.20 ± 0.83; Figure 1D), all of which showed significantly reduction in triptolide-treated group compared with untreated group (*p* < 0.05).

### Triptolide Reduces T Lymphocyte and Macrophage Infiltration and Inhibits mRNA Levels of Pro-Inflammatory and Pro-Fibrotic Cytokines in Allografts

**FIGURE 2:**
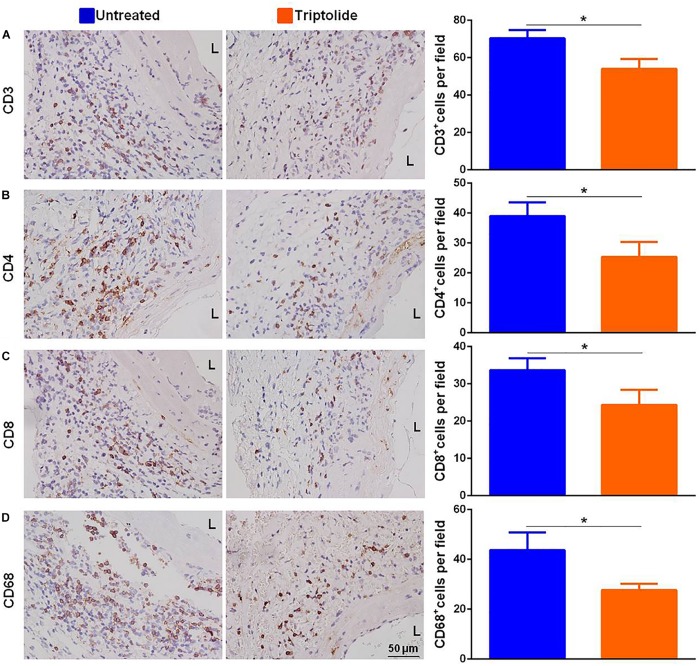
Triptolide decreases T lymphocyte and macrophage infiltration in allografts. Representative images and quantification of **(A)** CD3^+^, **(B)** CD4^+^, **(C)** CD8^+^, and **(D)** CD68^+^ cell infiltration in TV lesions. The positive cells were counted by automated cell counting using IPP 6.0 imaging software and expressed as the number of positive cells per field of a vascular section (400× magnification). Three independent experiments were performed and showed similar results. Data were expressed as mean ± SD. *p < 0.05 vs. untreated Student’s *t*-test. L, lumen.

**FIGURE 3:**
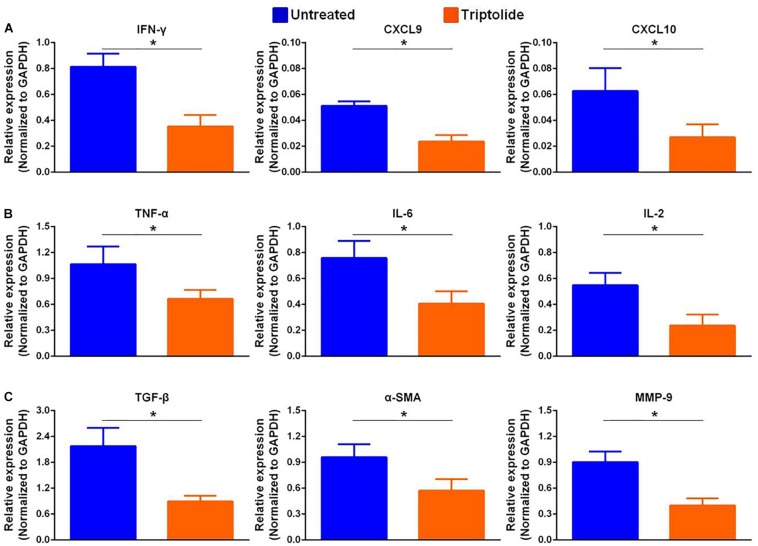
Triptolide inhibits mRNA expression levels of pro-inflammatory and pro-fibrotic factors. Aortic allograft animals were treated with or without triptolide for 4 weeks after transplantation. The mRNA expression of **(A)** *IFN*-γ and *IFN*-γ–inducible chemokines (*CXCL9* and *CXCL10*), **(B)** inflammatory chemokines (*TNF*-α, *IL-2*, and *IL-6*), and **(C)** pro-fibrotic factors (*TGF*-β, α*-SMA*, and *MMP-9*) were determined by RT-PCR. The mRNA levels were normalized to that of *GAPDH*. Three independent experiments were performed and showed similar results. Data were expressed as mean ± SD. *p < 0.05 vs. untreated Student’s *t*-test.

As shown in the representative images, the infiltration of CD3^+^, CD4^+^, CD8^+^, and CD68^+^ cells (Figures 2A–D) were significantly reduced in allografts following triptolide treatment (*p* < 0.05). Additionally, qRT-PCR analysis of the mRNA levels of *IFN*-γ and *IFN*-γ-inducing factors (C-X-C-motif chemokine ligand (*CXCL*)9 and *CXCL10*) (Figure 3A), pro-inflammatory chemokines [tumor necrosis factor (*TNF*)-α, interleukin (*IL*)-2, and *IL-6*)] (Figure 3B), pro-fibrotic factors (transforming growth factor (*TGF*)-β, α-smooth muscle actin (*SMA*), and matrix metalloproteinase (*MMP*)-9 (Figure 3C) revealed significantly reductions following triptolide treatment (*p* < 0.05).

### Triptolide Reduces the Number of IFN-γ-Producing T Lymphocytes *in vivo*

**FIGURE 4:**
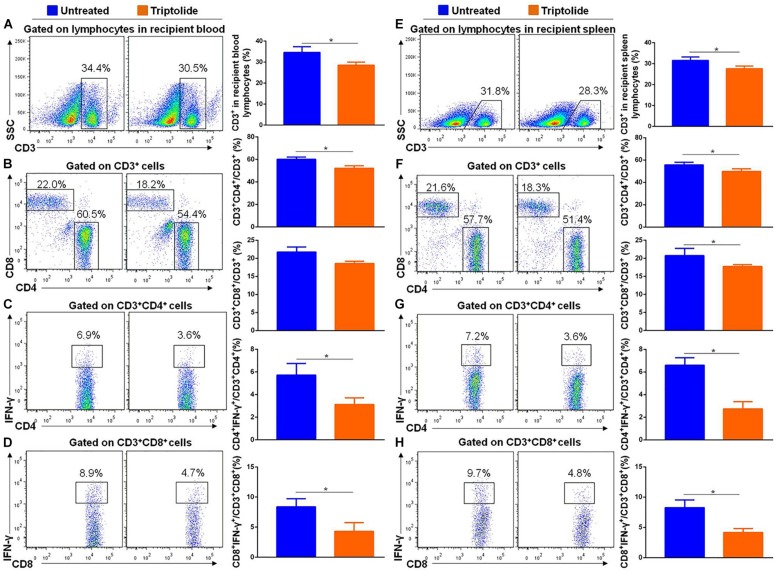
Triptolide reduces the number of IFN-γ–producing T lymphocytes in recipient blood and spleen. Aortic allograft animals were treated with triptolide or without for 4 weeks after transplantation. T cells in recipient blood and spleen were detected by flow cytometry. Representative dot plots, cell percentages, and counts of CD3^+^, CD3^+^CD4^+^, CD3^+^CD8^+^, CD4^+^IFN-γ^+^, and CD8^+^ IFN-γ^+^ T cells in recipient blood **(A–D)** and spleen **(E–H)** were shown. Data were represented as mean ± SD of at least three independent samples. *p < 0.05; Student’s *t*-test.

As shown in Figure 4, the results showed that triptolide significantly reduced CD3^+^ (34.6% ± 2.7% vs. 28.5% ± 1.5%), CD3^+^ CD4^+^ (60.2% ± 1.9% vs. 52.2% ± 2.2%), CD3^+^ CD8^+^ (20.8% ± 2.0% vs. 17.8% ± 0.5%), CD4^+^ IFN-γ^+^ (5.7% ± 1.0% vs. 3.1% ± 0.6%), and CD8^+^ IFN-γ^+^ (8.4% ± 1.4% vs. 4.3% ± 1.5%) T cells in recipient blood (*n* = 5 each; *p* < 0.05; Figures 4A–D). Similarly, in recipient spleen, triptolide also significantly reduced CD3^+^ (31.6% ± 1.6% vs. 27.6% ± 1.2%), CD3^+^ CD4^+^ (55.7% ± 2.3% vs. 49.8% ± 2.4%), CD3^+^ CD8^+^ (21.7% ± 1.4% vs. 18.6% ± 0.6%), CD4^+^ IFN-γ^+^ (6.6% ± 0.7% vs. 2.7% ± 0.7%), and CD8^+^ IFN-γ^+^ (8.3% ± 1.3% vs. 4.2% ± 0.7%) T cells (*n* = 5 each; *p* < 0.05; Figures 4E–H).

### Triptolide Inhibits IFN-γ Axis *in vitro*

**FIGURE 5:**
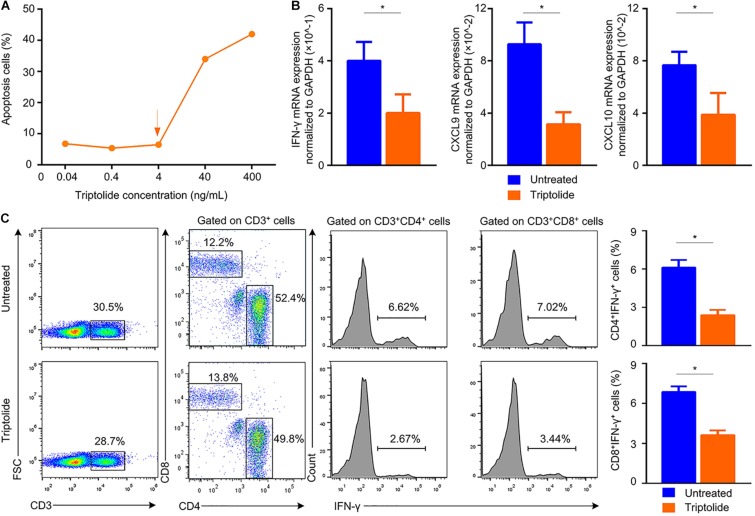
Triptolide reduces the mRNA expression of IFN-γ and IFN-γ–inducing factors, and also inhibits the frequency of IFN-γ–producing T lymphocytes *in vitro*. **(A)** Apoptotic cells were detected by flow cytometry. Mononuclear cells from the recipient spleens were incubated with various concentrations of triptolide (0.04, 0.4, 4, 40, and 400 ng/mL) for 72 h. To evaluate the effect of triptolide on IFN-γ-producing T cells *in vitro*, mononuclear cells from recipient spleens were stimulated with anti-CD3 plus anti-CD28 with or without tiptolide (4 ng/mL) at 37°C for 24 h. The mRNA expression of *IFN*-γ, *CXCL*9, and *CXCL10* was assessed by RT-PCR **(B)**. **(C)** Representative dot plots for frequencies and counts of IFN-γ–producing T cells in culture media were shown. Data were represented as mean ± SD of at least three independent samples. *p < 0.05; Student’s *t*-test.

Our results suggested that triptolide promoted lymphocyte apoptosis in a dose-dependent manner (Figure 5A). With triptolide at 4 ng/ml, the pro-apoptotic effect of triptolide was mild, while the mRNA levels of *IFN*-γ and *IFN*-γ-inducing factors (*CXCL9* and *CXCL10*) were significantly inhibited (*p* < 0.05; Figure 5B). Additionally, the levels of CD4^+^ IFN-γ^+^ and CD8^+^ IFN-γ^+^ cells in the triptolide-treated group were also significantly reduced (*p* < 0.05; Figure 5C), which was consistent with *in vivo* findings (Figure 4).

### Triptolide Decreases the Production of Donor-Specific Antibodies (DSAs) and Reduces the Amounts of B Cells and Plasma Cells *in vivo*

**FIGURE 6:**
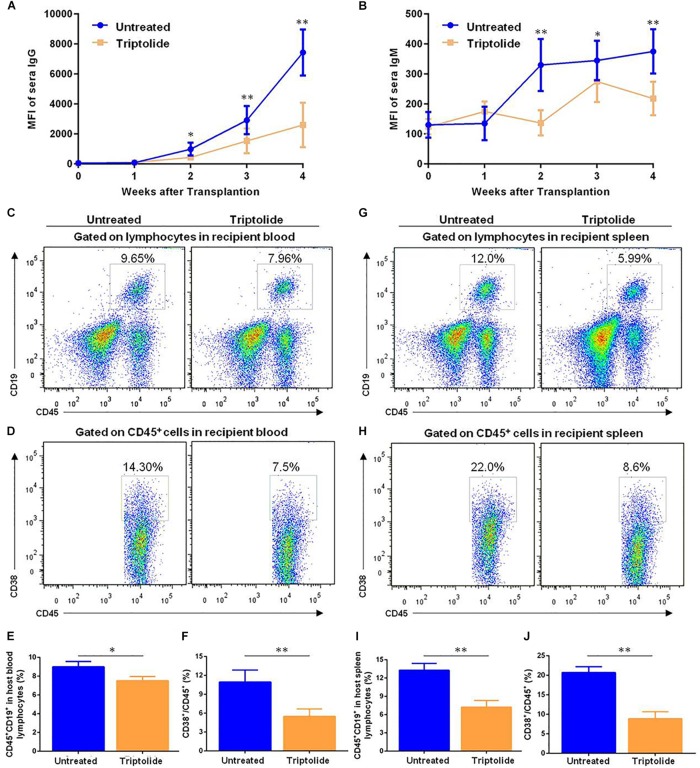
Triptolide decreases the production of donor-specific antibodies (DSA) and reduces the amounts of B cells and plasma cells *in vivo*. Transplant-recipient sera collected at the indicated time points were reacted with donor spleen cells and evaluated for antibody production by flow cytometry. Circulating levels of **(A)** IgG and **(B)** IgM are expressed as mean fluorescence intensity ± SD (*n* = 5). B cells (CD45^+^, CD19^+^) and plasma cells (CD45^+^, CD38^+^) were detected by flow cytometry in recipient blood and spleen 4 weeks after transplantation. Representative dot plots for frequencies and counts of B cells and plasma cells in recipient blood **(C–F)** and spleen **(G–J)** were shown. Data were represented as mean ± SD of at least three independent samples. ^∗^*p* < 0.05; ^∗∗^*p* < 0.01, Student’s *t*-test.

Other studies had revealed that DSA was related to a possible negative impact on the prognosis of TV (10, 11). However, it was unclear that whether triptolide could improve TV by inhibiting the levels of DSA. As shown in Figure 6, we found that the detection of IgG and IgM in triptolide-treated group were lower than those in untreated group (*p* < 0.05; Figures 6A,B). Flow cytometric analysis was applied to detect B cells (CD45^+^ CD19^+^) and plasma cells (CD45^+^ CD38^+^) in recipient blood, which revealed significant reductions of both B cells (9.00% ± 0.33% vs. 7.52 ± 0.26%) and plasma cells (12.91% ± 0.70% vs. 7.92 ± 0.25%) in the triptolide-treated group (*p* < 0.05; Figures 6C–F). In recipient spleen cells, triptolide also significantly reduced the amount of B cells (13.26% ± 0.66% vs. 7.23% ± 0.63%) and plasma cells (20.70% ± 0.88% vs. 8.87% ± 1.04%) (*p* < 0.05; Figures 6G–J).

### Triptolide Significantly Inhibits VSMC Migration Without Affecting VMSC Viability or Apoptosis

**FIGURE 7:**
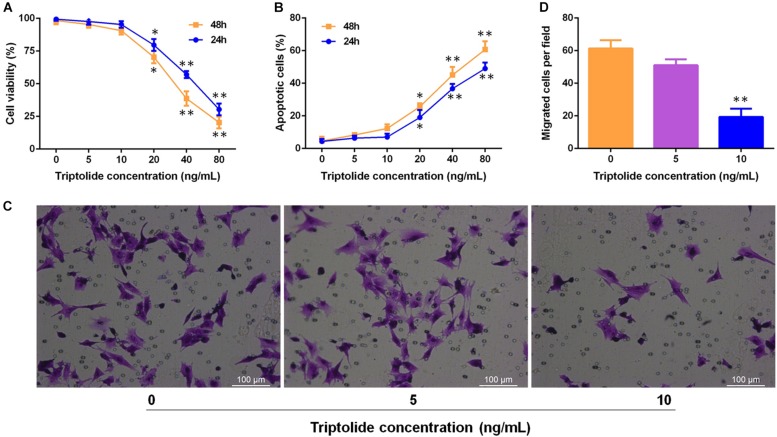
Triptolide significantly inhibits migration of VSMCs without affecting the viability and apoptosis of VSMCs. **(A)** Cell viability was assessed using the CCK-8 assay. **(B)** Cell apoptosis was detected by flow cytometry. **(C)** Cell migration was assessed through the Transwell migration assay. VSMCs were incubated in culture media containing various concentrations of triptolide (0, 5, and 10 ng/mL) for 24 h. Representative images of VSMCs that migrated through the gelatine to the lower-chamber side of Transwell membranes. **(D)** Quantification of the Transwell migration assay results. Data were represented as mean ± SD. Each experiment was performed three times. ^∗^*p* < 0.05, ^∗∗^*p* < 0.01 vs. untreated Student’s *t*-test.

To investigate the effects of triptolide on VSMC *in vitro*. VSMCs were incubated with different concentrations (*0, 5, 10, 20, 40, and 80* ng/mL) of triptolide for 24 h and 48 h. Then, the viability was assessed by the CCK8 assay (Figure 7A) and apoptosis was assessed by flow cytometry (Figure 7B). The data revealed that as the concentration of triptolide increased, cell viability decreased and cell apoptosis increased. With triptolide at 5 ng/mL or 10 ng/mL, no significant change in cell viability and apoptosis was found. We then administered triptolide at concentrations of 0, 5, and 10 ng/mL, which did not affect VSMC viability and apoptosis, to assess the effect of triptolide on VMSC migration using a transwell assay (Figures 7C,D). The results revealed significant reductions in VSMC migration in the triptolide-treated group (10 ng/mL).

## Discussion

The pathological features of transplant artery includes vascular inflammation, neointima formation and progressive luminal obstruction, which are consistent with the TV-related complications after solid organ transplantation, what’s more, compared with solid organ transplants such as heart and kidney, aortic transplant has the advantages of relatively simple operation. Therefore, many teams have used aortic transplantation as a small animal model for studying TV (25, 26). Our results provided strong evidence that triptolide significantly ameliorated pathological injury associated with TV through multiple pathways. Firstly, triptolide significantly reduced infiltration of inflammation cells and inhibited the levels of pro-inflammatory and pro-fibrotic cytokines in the graft. Secondly, triptolide decreased the number of B lymphocytes and plasma cells, as well as the levels of DSAs, in recipient. Thirdly, triptolide not only inhibited VSMC viability and promoted VSMC apoptosis but also significantly inhibited VSMC migration.

Triptolide has been widely studied for its extensive anti-inflammatory and anti-proliferative effects. Hachida and colleagues had demonstrated that triptolide inhibits the development of allograft vasculopathy via inhibition of PDGF-A signaling pathways in the rat heart transplantation (27). However, the authors only mentioned that triptolide improved allograft vasculopathy by inhibiting the proliferation of VSMC, without investigating the effect of triptolide on the migration of VSMC, or the anti-inflammatory effects of triptolide. VSMC is the main constituent cell of TV intima, and the migration of VSMC is considered the most critical factor associated with TV development (28). Here, we revealed that triptolide not only significantly decreased VSMC viability and increased VSMC apoptosis but also significantly inhibited VSMC migration. Importantly, we found that a lower concentration of triptolide inhibited VSMC migration in the absence of effects on cell viability or apoptosis.

Triptolide inhibited intimal thickening by suppressing the IFN-γ axis. Numerous studies had reported that triptolide inhibited the proliferation and activity of T lymphocytes (16, 29), and prevented the production of IFN-γ in some disease models (19, 30). However, no study investigated the effect of triptolide on the IFN-γ axis in attenuating TV. IFN-γ, which is the main cytokine secreted by T cells, exhibits crucial effect on TV development (8, 31). Loosdregt confirmed an increased expression of *IFN*-γ and IFN-γ-inducing factors (*CXCL9* and *CXCL10*) associated with TV (32). In our study, we detected that T lymphocytes infiltration, expression of *IFN*-γ, *CXCL9*, and *CXCL10*, and the amounts of IFN-γ-producing T lymphocytes in recipient were significantly decreased after triptolide treatment. Additionally, we explored the inhibitory effects of triptolide on the IFN-γ axis through a series of experiments *in vitro*. Therefore, our data showed that triptolide could not only inhibit the production of IFN-γ, but also inhibit the expression of IFN-γ-inducing factors (*CXCL9* and *CXCL10*) to attenuate the prognosis of TV.

Macrophages, pro-inflammatory and pro-fibrotic cytokines also play important roles in TV development (33–35). Although Crews and colleagues had described the ability of triptolide to inhibit chronic rejection in a rat kidney transplant model via inhibition of TGF-beta and VCAM-1 (36), the focus of this study was not TV, and the effects of triptolide in TV treatment were not fully demonstrated. In the murine aortic transplant model, our results showed that triptolide inhibited macrophages infiltration into grafts and significantly reduced the expressions of pro-inflammatory (*TNF*-α, *IL-2*, and *IL-6*) and pro-fibrotic factors (*TGF*-β, α*-SMA*, and *MMP-9*).

Previous studies confirmed that DSA levels were directly related to TV development (37). The effects of triptolide on antibodies production were not well studied, although two other studies on IgA nephropathy and lupus nephritis noted effects of triptolide on antibodies (38, 39). Our preliminary research had confirmed that triptolide reduced DSA levels in an acute rejection model (20). In the present study, we constructed a chronic rejection model by aorta transplantation and found that triptolide significantly reduced DSA levels. Additionally, the amounts of B lymphocytes and plasma cells in recipient were significantly decreased in the triptolide-treatment group. Our findings further confirmed that triptolide could also attenuate the prognosis of chronic rejection by inhibiting the production of DSA in the chronic rejection model.

Several limitations of this study should be noted. Firstly, triptolide has a wide range of effects and this study does not make further in-depth study on the mechanisms, especially, direct allospecific T cell response has not been demonstrated *in vitro*. Secondly, the mechanisms by which triptolide inhibits the migration of VSMC to improve the prognosis of TV remains to be further clarified. Finally, the concentration of triptolide used *in vivo* experiment is based on references and previous experience, so the toxic and side effects of triptolide are not described in the content.

In summary, our results reinforce the view that triptolide can significantly attenuate TV through inhibiting multiple pathways. These findings highlight the efficacy of triptolide in inhibiting TV and suggest triptolide as a potential ideal therapeutic strategy of great clinical value for preventing TV-related complications and improving the long-term survival of transplant recipients.

## Data Availability Statement

The datasets generated for this study are available on request to the corresponding author.

## Ethics Statement

The animal study was reviewed and approved by the Sun Yat-sen University Institutional Ethical Guidelines.

## Author Contributions

ZL: study design and drafting of the manuscript. TL and ZL: performed the transplantation model. YZ and FH: performed the experiments. QS and HZ: pathological scoring. ZY and ZL: statistical analysis; QS: conceived the study and critical revision of the manuscript.

## Conflict of Interest

The authors declare that the research was conducted in the absence of any commercial or financial relationships that could be construed as a potential conflict of interest.
